# Antimicrobial consumption in three pediatric and neonatal intensive care units in Saudi Arabia: 33-month surveillance study

**DOI:** 10.1186/s12941-019-0320-2

**Published:** 2019-07-03

**Authors:** Hanan H. Balkhy, Aiman El-Saed, Ali AlShehri, Mohammad Alshaalan, Omar Hijazi, Ashraf El-Metwally, Sameera M. Aljohany, Saif Al Saif

**Affiliations:** 1 King Saud Bin Abdulaziz University for Health Sciences, Riyadh, Saudi Arabia; 2 King Abdullah International Medical Research Center, Riyadh, Saudi Arabia; 3 Infection Prevention and Control Department, King Abdulaziz Medical City (KAMC), Ministry of National Guard Health Affairs (MNGHA), P.O. Box 22490, Riyadh, 11426 Saudi Arabia; 4 Community Medicine Department, Faculty of Medicine, Mansoura University, Mansoura, Egypt; 5 Pediatric Intensive Care, King Abdullah Specialized Children’s Hospital, MNGHA, Riyadh, Saudi Arabia; 6 Department of Pediatrics, Infectious Disease Division, KAMC, MNGHA, Riyadh, Saudi Arabia; 7 Pediatric Cardiothoracic Intensive Care Unit, KAMC, MNGHA, Riyadh, Saudi Arabia; 8 Pathology and Laboratory Medicine Department, KAMC, MNGHA, Riyadh, Saudi Arabia; 9 Neonatal Intensive Care, King Abdullah Specialized Children’s Hospital, MNGHA, Riyadh, Saudi Arabia

**Keywords:** Antibiotics, Defined daily dose, Days of therapy, Children, Saudi Arabia

## Abstract

**Background:**

Despite their critical role in antimicrobial stewardship programs, data on antimicrobial consumption among the pediatric and neonatal population is limited internationally and lacking in Saudi Arabia. The current study was done as part of our antimicrobial stewardship activities.

**Objectives:**

To calculate overall and type-specific antimicrobial consumption.

**Methods:**

A prospective surveillance study was conducted at King Abdulaziz Medical City, Riyadh, Saudi Arabia, between October 2012 and June 2015 in two pediatric and one neonatal intensive care units (ICUs). Consumption data were collected manually on a daily basis by infection control practitioners. Data were presented as days of therapy (DOT) per 1000 patient-days and as frequency of daily consumption.

**Results:**

During the 33 months of the study, a total of 30,110 DOTs were monitored during 4921 admissions contributing 62,606 patient-days. Cephalosporins represented 38.0% of monitored antimicrobials in pediatric ICUs followed by vancomycin (21.9%), carbapenems (14.0%), aminoglycosides (8.8%), and piperacillin/tazobactam (8.8%). Their consumption rates were 265.1, 152.6, 97.6, 61.4, and 61.4 DOTs per 1000 patient-days (respectively). Aminoglycosides represented 45.4% of monitored antimicrobials in neonatal ICU followed by cephalosporins (30.4%) vancomycin (13.6%), and carbapenems (8.3%). Their consumption rates were 147.5, 98.7, 44.3, and 27 DOTs per 1000 patient-days (respectively).

**Conclusion:**

Cephalosporins are frequently used in pediatric ICU while aminoglycosides are frequently used in neonatal ICU. The local consumption of cephalosporins and carbapenems in both ICUs is probably higher than international levels. Such data can help in establishing and monitoring the functions of antimicrobial stewardship activities aiming to ensure judicious consumption of antimicrobials.

## Background

The continuous rise of antimicrobial resistance (AMR) has led to its recognition as a global public health threat. Highly resistant pathogens are more frequently recognized and have created challenges for physicians to effectively treat serious human infections [1, 2]. In Saudi Arabia, extended-spectrum beta-lactamase (ESBL) producing enterobacteriaceae and carbapenem-resistant and multidrug-resistant *Pseudomonas aeruginosa* and *Acinetobacter baumannii* are highly prevalent [3, 4]. While several factors are recognised as contributing to the increasing AMR rates, the extensive and/or inappropriate consumption of antimicrobial agents is considered a major factor for AMR in humans [5]. It has been estimated that between 33 and 78% of hospitalized children are receiving at least one antibiotic [6]. The extensive consumption of antimicrobials among hospitalized children may be related to the considerable resistant healthcare-associated infections they are facing [7]. Additionally, several pediatric and neonatal infections are among the top candidates of inappropriate antimicrobial consumption [8, 9]. As in many parts of the world, the majority of antimicrobial prescriptions at healthcare and community setting in Saudi Arabia are probably inappropriate; specially empirically-prescribed and broad-spectrum antimicrobials [10]. In addition to the increasing burden of resistant infections, this inappropriate antimicrobial consumption is driven by diagnostic uncertainty, fragmented medical care, limited time allocated for physician–patient interaction, and physician factors such as complacency, fear of failure, and lack of expertise [11].

Pediatric-specific antimicrobial stewardship programs (ASP) are increasingly implemented to decrease antimicrobial consumption, antimicrobial costs, and prescription errors and adverse events [6, 12]. For example, with the guidance of the Transatlantic Taskforce on AMR (TATFAR) [6], 31 out of 42 pediatric hospitals in the USA either have already established ASPs or are in the process of initiating ones [13]. Similarly, our hospital initiated a stepwise ASP to cover both pediatric and adult populations. One of the major challenges was the lack of pediatric-specific data on antimicrobial consumption, both locally and nationally. Since resources are limited, such data would be critical in prioritizing interventions for the ASP in our organization. Previous data quantifying antimicrobial consumption in Saudi Arabia focused on overall hospital consumption with no pediatric/adult distinction [14, 15]. The aim of the current study is to calculate overall and type-specific antimicrobial consumption for pediatric and neonatal ICUs.

## Methods

### Setting

The current study was conducted at King Abdulaziz Medical City-Riyadh (KAMC-R), Ministry of National Guard Health Affairs (MNGHA) in Saudi Arabia. This healthcare system is governmentally funded and provides healthcare services for about 750,000 Saudi National Guard soldiers, employees and their families. The care provided ranges from primary and preventive care to tertiary care. The three ICUs included in the current study were pediatric general medical/surgical ICU (20 beds), pediatric cardiothoracic ICU (12 beds), and neonatal ICU (40 beds). The three ICUs provide care for approximately 1850 patients per year, spending approximately 23,300 patient-days per year. The overall bed utilization in the included ICUs was 94%.

### Population

For the numerator, all patients < 16 years admitted to one of the included ICUs during the study period who received at least one dose of any of the included antimicrobials (shown below). For the denominator data, all patients < 16 years admitted to one of the included ICUs during the study period irrespective of antimicrobial consumption. Exclusion criteria, age ≥ 16 years, prescriptions of antimicrobials not included in the study, and prescriptions of antimicrobial by route other than parenteral or oral routes.

### Study design

A prospective surveillance study at King Abdulaziz Medical City, Riyadh, Saudi Arabia, between October 2012 and June 2015. The study was approved by the King Abdullah International Medical Research Center (KAIRMC) ethical committee and was funded by the KAIRMC.

### Outcome definition

Days of therapy (DOT) were defined as the aggregate sum of days (including admission and discharge days) for which any amount of a specific antimicrobial agent was administered to individual patients [16]. Patient days were calculated as the number of patients who were present for any portion of each day (including admission and discharge days) of a calendar month at a specific ICU. Antimicrobials frequently used in treatment of healthcare-associated infections or locally monitored for resistance patterns were included; aminoglycosides (amikacin or gentamicin), carbapenems (imipenem or meropenem), cephalosporins (ceftriaxone, cefotaxime, ceftazidime, or cefepime), fluoroquinolones (ciprofloxacin or norfloxacin), piperacillin/tazobactam, vancomycin, colistin, caspofungin, and amphotericin B.

### Data collection

Antimicrobial agents prescribed to pediatric/neonatal patients were collected prospectively on a daily basis by infection control practitioners, using specially customized data entry file for the study. The following variables were recorded; age, gender, ICU type; and name, dose, frequency, and route of antimicrobial consumption. Antimicrobial event was recorded (as a new row in the data file) once a patient received at least one dose of one of the included antimicrobials during a certain day. Each antimicrobial event (row) was considered as 1 day of therapy. The same patient can contribute to more than one antimicrobial event on the same day if he/she received more than one antimicrobial. The same patient can contribute to more than one specific antimicrobial event during different days if he/she received the same antimicrobial for more than 1 day.

### Statistical methods

Continuous variables were presented as means and standard deviations as well as sum. Categorical variables were presented as frequencies and percentages. Antimicrobial consumption were presented as DOTs per 1000 patient days and per 100 admissions. SPSS (Version 23.0. Armonk, NY: IBM Corp) was used for all statistical analyses.

## Results

During the 33 months of the study, a total 30,110 DOTs were monitored for 4921 admissions contributing 62,606 patient-days. The average admission duration was 12.7 days (ranged between 8.5 in pediatric ICUs and 20.0 in neonatal ICU). The average age was 1.54 ± 2.99 years (ranged between 0 and 16 years). As shown in Table 1, the majority of antimicrobial consumption was observed in infants (73.5%). Two pediatric ICUs contributed to 60.9% of the data while a single neonatal ICU contributed for the rest of data. Based on the sum of DOTs, the top 5 most prescribed antimicrobials included vancomycin (18.6%), ceftazidime (18.0%), cefotaxime (12.2%), amikacin (12.0%), and meropenem (11.8%). Norfloxacin, imipenem, and cefepime were almost not prescribed in pediatric and neonatal ICUs. Almost all (99.9%) antimicrobial consumption was through intravenous route.

**Table 1:** Overall antimicrobial consumption by the patient demographics, ICU type, and type of antimicrobials prescribed

	Sum of DOT during the study	
	Value	%
Overall	30,110	100.00
Age		
First month	10,311	34.2
1–12 months	11,822	39.3
1–16 years	7977	26.4
Gender		
Male	16,076	53.4
Female	14,020	46.6
Type of ICU		
Pediatric general	10,249	34.0
Pediatric cardiothoracic	8075	26.8
Neonatal	11,786	39.1
Antimicrobials		
Amikacin	3608	12.0
Gentamicin	3361	11.2
Imipenem	4	0.0
Meropenem	3543	11.8
Ceftriaxone	1411	4.7
Cefotaxime	3671	12.2
Ceftazidime	5427	18.0
Cefepime	43	0.1
Ciprofloxacin, IV	632	2.1
Ciprofloxacin, oral	14	0.0
Norfloxacin, IV	1	0.0
Piperacillin/tazobactam	1654	5.5
Vancomycin, IV	5615	18.6
Vancomycin, oral	5	0.0
Colistin	264	0.9
Caspofungin	431	1.4
Amphotericin B	426	1.4
Route		
Intravenous	30,084	99.9
Oral	26	0.1

Table 2 shows ICU-specific antimicrobial consumption in DOTs per patient-days and admissions. The overall antimicrobial consumption during the study was 480.9 per 1000 patient-days. The patients were using approximately 6.1 DOTs of one or more antimicrobials during a single admission. The antimicrobial consumption during the same admission was higher at neonatal ICU compared with pediatric ICUs (650.1 versus 589.6 DOTs per 100 admissions). However, after adjusting for the admission duration, patients at pediatric ICUs had double days of antimicrobial consumption compared patients at neonatal ICU (697.0 versus 324.5 DOTs per1000 patient-days).

**Table 2:** ICU and type specific antimicrobial consumption in DOTs, KAMC-R, 2012–2014

	Pediatric general	Pediatric cardiothoracic	Pediatric subtotal	Neonatal	Total
Denominator					
Patient days	14,469	11,820	26,289	36,317	62,606
Admissions	1616	1492	3108	1813	4921
Rates per 1000 patient-days					
Aminoglycosides	38.5	89.4	61.4	147.5	111.3
Carbapenems	85.8	111.9	97.6	27.0	56.7
Cephalosporins	237.3	299.2	265.1	98.7	168.5
Fluoroquinolones	23.8	19.7	22.0	1.9	10.3
Piperacillin/tazobactam	91.8	24.2	61.4	1.1	26.4
Vancomycin	185.8	112.0	152.6	44.3	89.8
Colistin	12.3	5.9	9.4	0.4	4.2
Caspofungin	18.2	10.7	14.8	1.1	6.9
Amphotericin B	14.8	10.2	12.7	2.5	6.8
Total	708.3	683.2	697.0	324.5	480.9
Rates per 100 admissions					
Aminoglycosides	34.5	70.8	51.9	295.4	141.6
Carbapenems	76.9	88.7	82.5	54.2	72.1
Cephalosporins	212.4	237.0	224.2	197.6	214.4
Fluoroquinolones	21.3	15.6	18.6	3.8	13.1
Piperacillin/tazobactam	82.2	19.2	51.9	2.2	33.6
Vancomycin	166.3	88.7	129.1	88.7	114.2
Colistin	11.0	4.7	8.0	0.9	5.4
Caspofungin	16.3	8.4	12.5	2.3	8.8
Amphotericin B	13.2	8.0	10.7	5.1	8.7
Total	634.2	541.2	589.6	650.1	611.9
Average DOT per admission	6.3	5.4	5.9	6.5	6.1

For the most frequently prescribed antimicrobials in pediatric ICUs, the average consumption of cephalosporins, vancomycin, carbapenems, aminoglycosides, and piperacillin/tazobactam were 265.1, 152.6, 97.6, 61.4, and 61.4 DOTs per 1000 patient-days (respectively) and 224.2, 129.1, 82.5, 51.9, and 51.9 DOTs per 100 admissions (respectively). For the most frequently prescribed antimicrobials in neonatal ICU, the average consumption of aminoglycosides, cephalosporins, vancomycin, and carbapenems were 147.5, 98.7, 44.3, and 27 DOTs per 1000 patient-days (respectively) and 295.4, 197.6, 88.7, and 54.2 DOTs per 100 admissions (respectively).

**Fig. 1:**
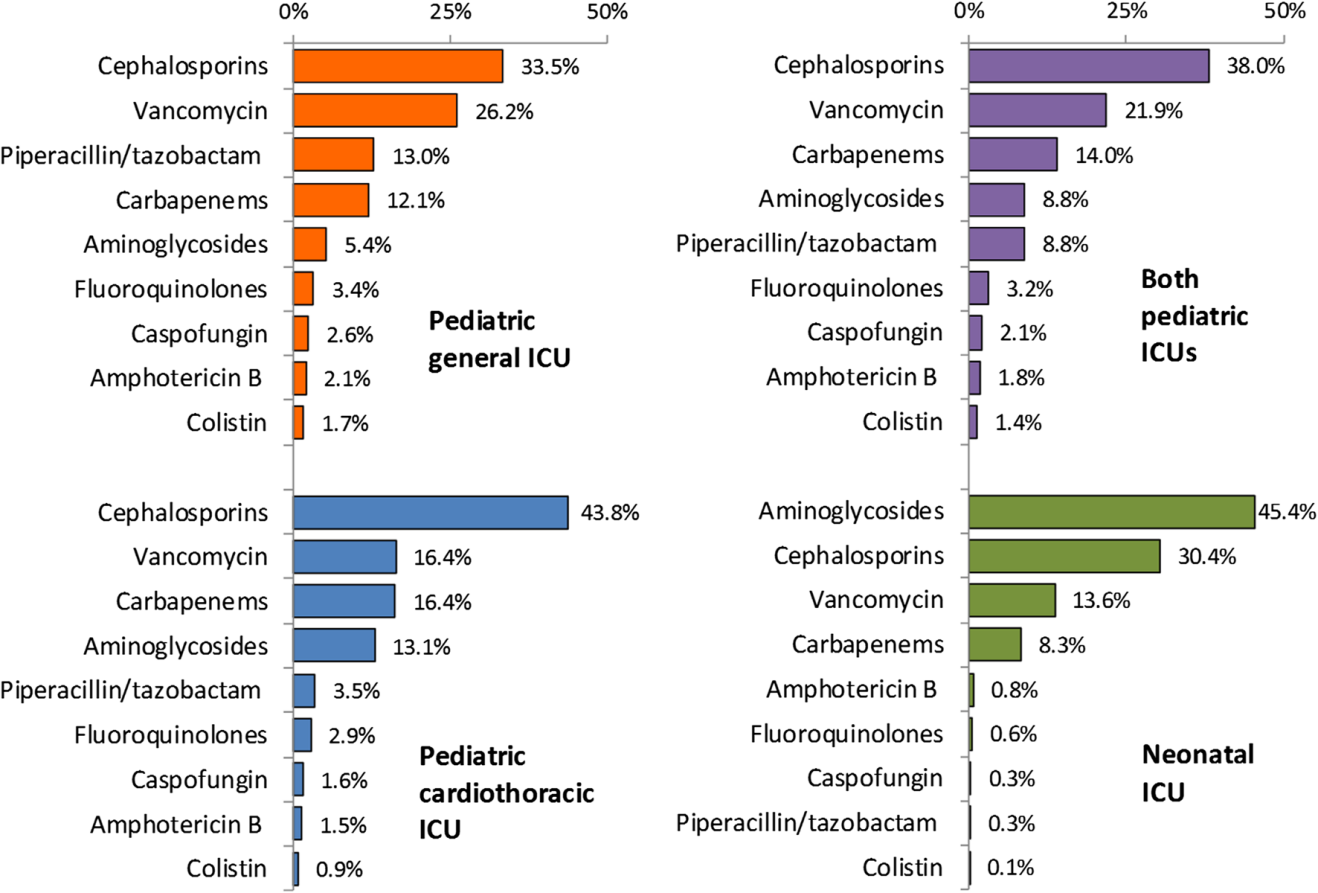
Frequency of daily consumption of different antimicrobials (relative to total DOTs) in pediatric and neonatal ICUs

Figure 1 shows the frequency of daily consumption of different antimicrobials (relative to total DOTs). Cephalosporins represented 38.0% of daily consumption of all antimicrobials in pediatric ICUs followed by vancomycin (21.9%) and carbapenems (14.0%). Vancomycin and piperacillin/tazobactam were prescribed more frequently in patients at pediatric general ICU while carbapenems were more frequently prescribed in patients at pediatric cardiothoracic ICU. Aminoglycosides represented 45.4% of daily consumption of all antimicrobials in neonatal ICU followed by cephalosporins (30.4%) and vancomycin (13.6%). Unlike pediatric ICUs, piperacillin/tazobactam and fluoroquinolones were rarely prescribed in neonatal ICU.

**Fig. 2:**
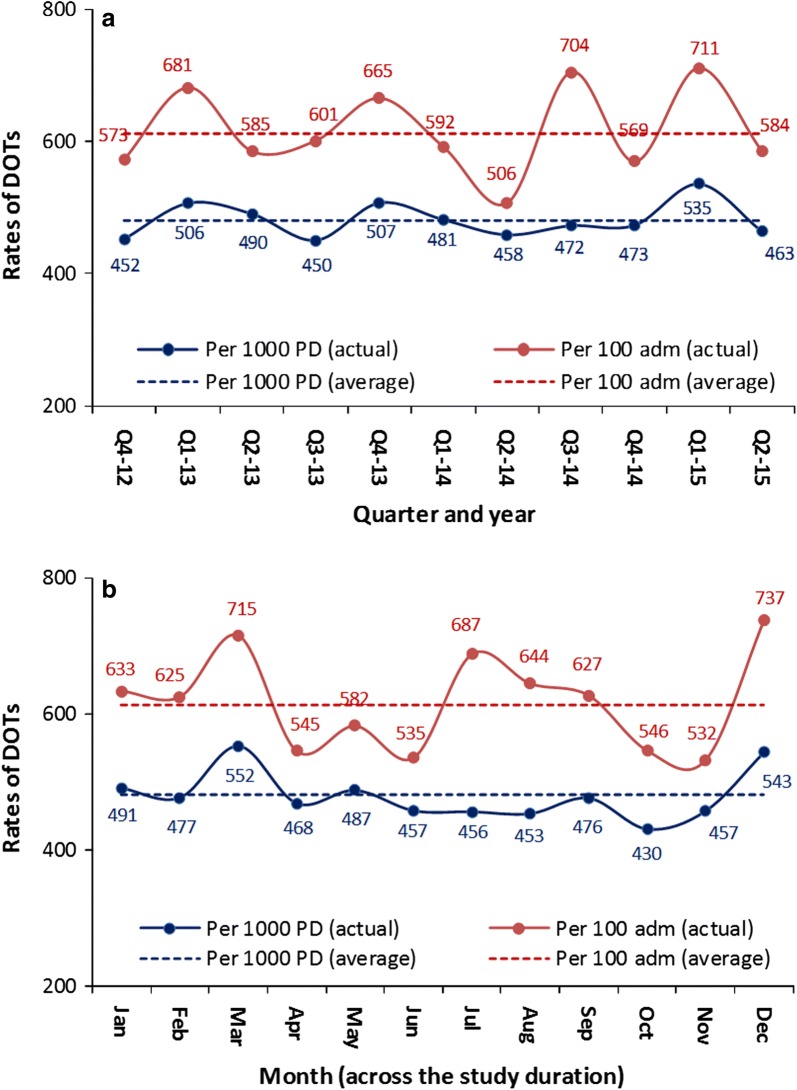
Trends (**a**) and seasonality (**b**) of antimicrobial consumption in pediatric and neonatal ICUs in DOTs per 1000 patient-days and per 100 admissions

Figure 2 shows the trends and seasonality of antimicrobial consumption. There was some variability in the trends and seasonality that were more apparent in DOTs per admissions than DOTs per patient-days. Three peaks of antimicrobial consumption were identified; in March, July, and December.

## Discussion

We are reporting the levels of pediatric and neonatal antimicrobial consumption at ICU setting at a major tertiary care hospital in Saudi Arabia. There has been a substantial debate about the best metric to estimate antimicrobial consumption [17]. Compared to defined daily dose (DDD), DOTs has been suggested as a better metric in pediatric populations, as they are likely to adjust for the differences in antimicrobial consumption because of age and weight modifications of antimicrobial doses compared with adults [17, 18]. However, DOTs may overestimate overall antimicrobial consumption in case of polymicrobial consumption and is not suitable to monitor dose-dependent appropriateness of antimicrobial consumption [19]. Prescribed daily dose has been also suggested as an appropriate metric for antimicrobial consumption in neonatal and pediatric populations [18, 20]. However, it involves collection of drug dose data with identical findings to DOT method [18].

Benchmarking the current (overall and type-specific) antimicrobial consumption is challenging because of the limited data on antimicrobial consumption in neonatal and pediatric patients internationally and absolute lack of such data locally. Additionally, DDDs were the used metric in all non-recent data, which could be misleading in case of comparing different case mix [21, 22]. Furthermore, most DOTs data were not ICU-specific nor differentiating between different types of pediatric ICUs [23–25]. Nevertheless, using DOTs per 1000 patient-days, the overall antimicrobial consumption in pediatric ICUs in the current study (697.0) was generally comparable to those reported in USA and Canada (approximately 750) [25, 26]. In contrast, the overall antimicrobial consumption in neonatal ICU in the current study (324.5) was slightly higher than reported in USA (approximately 250) [9, 27] but much lower than reported in Russia (> 1000) [28].

The current findings showed high consumption of cephalosporins, vancomycin and carbapenems in pediatric ICUs and aminoglycosides and cephalosporins in a neonatal ICU. For pediatric ICUs, the consumption of cephalosporins and carbapenems in the current study was higher while the consumption of vancomycin was comparable to the levels reported in USA and Canada [18, 26]. For neonatal ICUs, the consumption of aminoglycosides was comparable while consumption of cephalosporins and carbapenems were much higher than reported in the USA [9, 27].

Although the underlying causes were not examined, the higher consumption rates of cephalosporins and carbapenems in both pediatric and neonatal ICUs observed in the current study need to be assessed for their appropriateness. In a review of antimicrobial misuse in Saudi Arabia, children were the main focus and the rates of inappropriateness ranged between 41 and 92% [10]. Additionally, in a recent study that reviewed pediatric and adult prescriptions at the emergency department at our hospital, children were at higher risk of inappropriate antimicrobial prescriptions than adults (58% versus 39%), mainly in the form of inappropriate duration and/or dosage [29]. The burden of inappropriate antimicrobial prescriptions in that study was highest with cephalosporins and in respiratory and middle ear infections [29]. However, these high rates of antimicrobial inappropriateness may not be fairly projected to our population as these studies were done in non-ICU setting [10, 29]. Furthermore, previous work done in our neonatal ICU showed much lower rates of antimicrobial inappropriateness (10%), with vancomycin, third generation cephalosporins and carbapenems were the most inappropriately used ones [30].

Irrational consumption of broad spectrum antibiotics is a key factor in promoting AMR [5]. This may be responsible for the high burden of multidrug-resistant organisms specially ESBL and carbapenemase-producing Gram-negative bacteria reported in Saudi Arabia and adjacent countries [3, 4]. The current findings re-emphasize the importance of setting efficient ASP programs [6, 12]. Local successful experiences are promising. For example, simple restriction of specific antimicrobials as a part of stepwise implementation of ASP in one of the local hospitals was associated with reduction of antimicrobial utilization without increasing mortality [31]. While it could be more effort and time consuming, prospective audit and feedback as a part of proactive ASP program in another local hospital was successful in reducing antimicrobial utilization and improving the appropriateness of empirical antibiotics [32]. However, building a dedicated healthcare is still a local challenge [33].

The current study is the first local study to address antimicrobial consumption among pediatric and neonatal critical care patients. It included several frequently used antimicrobials and measured the consumption using DOTs for better benchmarking. Nevertheless, we acknowledge a number of limitations; the interpretation of the current data would have been more useful if combined with data on the appropriateness, degree of implementation of local protocols of antimicrobial consumption, culture-dependent de-escalation therapy, predominant bacterial resistance patterns, and patient mix. Additionally, being a single center study, the findings of the current study should be interpreted with caution. However, we believe that the current data can be perfectly used in internal and external benchmarking. Furthermore, it can be used as a baseline to monitor long term impacts of ASP activities and can potentially help activities aiming to improve prescription practices.

In conclusion, we are reporting high consumption of cephalosporins in pediatric ICUs and aminoglycosides in a neonatal ICU at a tertiary care hospital in Saudi Arabia. The local consumption rates of cephalosporins and carbapenems in both pediatric and neonatal ICUs are probably higher than international levels. Such data will be of value in establishing and monitoring the functions of ASP activities aiming to ensure judicious consumption of antimicrobials. Additionally, providing treating physicians and other stakeholders with feedback on the levels of antimicrobial consumption in their units is expected to reduce the amount of antimicrobial consumption and probably resistance [34, 35].

## Data Availability

The datasets used and/or analyzed during the current study are available from the corresponding author on reasonable request.
